# Analysis of clinical and pathological prognostic factors of survival in rectal adenocarcinoma treated with preoperative radiochemotherapy

**DOI:** 10.1590/acb401125

**Published:** 2025-02-10

**Authors:** Sarhan Sydney Saad, Nora Forones, Gaspar Lopes, Jaques Waisberg, Elesiario Caetano, Ricardo Artigiani-Neto, Delcio Matos

**Affiliations:** 1 Universidade Federal de São Paulo – Escola Paulista de Medicina – Departamento Cirurgia – São Paulo (SP) – Brazil.; 2 Universidade Federal de São Paulo – Escola Paulista de Medicina – Departamento Medicina – São Paulo (SP) – Brazil.; 3 Universidade Federal de São Paulo – Escola Paulista de Medicina – Departamento Patologia – São Paulo (SP) – Brazil.

**Keywords:** Rectal Neoplasms, Chemoradiotherapy, Survival Analysis

## Abstract

**Purpose:**

To identify the prognostic variables related to the survival of patients operated on for adenocarcinoma of the rectum who underwent preoperative radiochemotherapy (RCT).

**Methods:**

We studied 70 patients from the Discipline of Surgical Gastroenterology at Escola Paulista de Medicina from 2000 to 2019, with rectal cancer located up to 10 cm from the anal verge and with stages II or III, submitted to preoperative RCT and curative surgery (R0) and with follow-up of at least 12 months. Clinical restaging was performed four to six weeks after the end of neoadjuvant treatment to characterize the degree of clinical tumor regression. Surgery by laparotomy or videolaparoscopy was performed six to 12 weeks after RCT. Primary endpoint were: overall survival (OS), disease-free survival (DFS), metastasis-free survival (MSS), and neoplasm-specific survival (SEN). These were compared with gender, age, carcinoembryonic antigen (CEA) dosage, distance from the tumor to the anal verge, radiation dose, radiotherapy-surgery interval, clinical regression, type of surgery, pT and pN TNM stage tumor, number of nodes, circumferential resection margin, and complete pathological response. Survival was assessed by Kaplan-Meier curves. Univariate and multivariate Cox analyses were calculated to identify factors associated with survival outcomes.

**Results:**

The mean follow-up time was 62 months. The pathological complete response rate was 18.6%. Univariate cox regression showed a significant relationship of CEA equal to or greater than 4 ng/mL with DFS and MFS, pT3/pT4 staging with DFS, MFS and SEN, pN1/N2 with DFS, MFS and SEN and stages II and III with DFS and MFS. Multivariate regression found that CEA, pT, and pN staging are independent prognostic factors for DFS, MFS, and SEN.

**Conclusion:**

Carcinoembryonic antigen level prior to radiotherapy, pT staging and pN staging were independent prognostic factors for survival in patients with rectal adenocarcinoma who are treated with preoperative radiochemotherapy.

## Introduction

Colorectal cancer is a highly prevalent malignancy. The estimated number of new colorectal cancer cases in the United States of America in 2022 would be 151,030^1^. In 44,850 of these cases, the malignancy would be located in the rectum, and the expected mortality is 52,580 people.

In Brazil, as predicted by the Instituto Nacional de Câncer^2^, for the year 2022, 45,630 new cases of colorectal cancer were expected. In 2020, there were 20,245 deaths.

The treatment of rectal cancer is based primarily on surgery. The local recurrence of the malignancy ranges from 20 to 30% and is an indicator of the success of the surgical treatment since most of the time the recurrence is surgically unresectable^3^.

Radiation therapy and chemotherapy are part of the treatment of rectal cancer and aim to reduce the risk of local and distant recurrence and thus increase the time of disease-free survival and reduce the mortality caused by this type of cancer^4^.

Preoperative radiation therapy enables the reduction of tumor volume and, therefore, safer curative surgical resection, decreasing the chances of leaving inadequate circumferential margins. The absence of surgical manipulation maintains the tumor tissue’s blood supply and oxygenation level is preserved, making it up to three times more sensitive than tissue with fibrosis. Sterilization of tumor cells from perirectal tissues and malignant cell aggregates in the lymphatic ducts and tissue spaces reduce tumor spread and prevent local recurrence. In the postoperative period of rectal abdominoperineal resection, there is some risk of actinic complications in the small intestine. Obtaining a distal margin to the tumor may enable sphincter preservation surgery instead of resection of the rectum^5^^–^ 9.

The disadvantages are related to the loss of reliability of the pathology staging due to the changes promoted by radiation and the occasional use of radiation therapy in patients with initial rectal tumors who would not need this type of treatment^10^.

Because of the better anatomical knowledge of the dissemination pathways of rectal cancer, represented by the mesorectum, associated with the good results achieved with radiation therapy associated with preoperative chemotherapy, there was an improvement in curative resection rates for rectal cancer, as well as a significant decrease in the rates of local recurrence, indicating better local control of the malignancy^11^^–^ 14.

Despite this better local control of the malignancy, many authors have tried to find prognostic markers of response to the radiochemotherapy (RCT) treatment. These predictive factors could predict patients at higher risk of local recurrence and those with better results in terms of survival. The studied markers are based on the patient’s clinical characteristics, the distance of the tumor from the anal verge, clinical staging, radiation therapy or chemotherapy protocol, degree of tumor regression achieved with the treatment, pathology staging, and molecular blood, histopathological and genetic markers. With these markers, one could predict the possibility of a tumor’s complete pathological response to radiation therapy, the chance of local tumor recurrence, and disease progression, thus helping both patients and physicians. The knowledge of these markers after neoadjuvant treatment helps the attending physician better predict the patient’s prognosis and make the follow-up with greater care.

The objective of this study was the identification of prognostic factors related to the survival of patients with rectal adenocarcinoma undergoing preoperative radiation therapy and chemotherapy and surgical treatment.

## Methods

The selected cases were patients with rectal cancer treated by the Discipline of Surgical Gastroenterology of the Escola Paulista de Medicina of the Universidade Federal de São Paulo (Unifesp) from 2012 to 2018.

The research was approved by the Research Ethics Committee of Escola Paulista de Medicina of Unifesp after the submission of the project to the National Commission for Research Ethics of the Ministry of Health (National Health Council – Resolution CNS no. 196/96), according to opinion no. 66.318.

### Research methodology

The study was conducted with 70 rectal cancer patients who underwent neoadjuvant treatment followed by surgery from November 2012 to February 2018 and followed up for at least 12 months.

Rectal cancer with biopsy of the adenocarcinoma located up to 10 cm from the anal verge with stage II or III in the classification of the American Joint Committee on Cancer^15^, with treatment that included preoperative radiation therapy and chemotherapy, followed by curative surgery (R0) with follow-up of at least 12 months. Patients who were unable to complete neoadjuvant treatment for clinical reasons and those in whom palliative surgery was performed (R1 or R2) were excluded, as well as those with synchronous colorectal cancer, cancer in other locations, previous radiation therapy and chemotherapy treatment or those with distant metastases.

The initial clinical staging included clinical examination, laboratory tests, carcinoembryonic antigen (CEA), proctological examination, colonoscopy, and computed tomography (CT) of the chest, abdomen, and pelvis. The radiation therapy used three (posteroanterior and latero-lateral) or four (posteroanterior, anteroposterior, and latero-lateral) irradiation fields according to the dose distribution in the target volume and normal tissues, with a dose of 45 Gy and boost of 5.4 Gy, completing a total dose of 50.4 Gy. The treatment was administered daily, five times a week, for five weeks, with a fraction of 1.8 Gy per day, with weekly assessment. The chemotherapy regimen provided for the bolus delivery of 5-fluorouracil (5-FU) at the dosage of 325 mg/m^2^/day and folinic acid at the dosage of 20 mg/m^2^/day at the first and last week of radiation therapy. Except for patients with complete pathological responses, all patients received postoperative chemotherapy treatment. The drugs and dosages used were the same, starting four to six weeks after surgery and administered once a week for six months. During chemotherapy treatment, patients were assessed every two weeks with clinical examination and laboratory analysis.

Clinical restaging was done four to six weeks after the end of the neoadjuvant treatment. The new clinical staging was done to discover the degree of tumor regression.

To assess the clinical response to the neoadjuvant treatment, a regression scale was created based on the change in lesion size, distance from the anal verge, and mobility, which was stratified into five grades, as described below:

- Grade 0: no change;
- Grade 1: regression up to 25%;
- Grade 2: regression from 25 to 50%;
- Grade 3: regression from 50 to 75%;
- Grade 4: regression greater than 75%.

Surgeries were performed at intervals that ranged from six to 12 weeks, using laparotomy or laparoscopy. All of them observed the mesorectum dissection rules described by Heald et al.^16^. Three types of operative procedures were performed: local resection, lower anterior resection with colorectal anastomosis or rectal abdominoperineal resection. The option of local resection of the rectal lesion was made when the patient did not accept a definitive colostomy or when the surgeon, upon finding a small lesion in the rectum with a negative previous biopsy, performed a complete excisional biopsy of the lesion. The operative procedure of this excision always followed the technical principles of local resection of rectal cancer, with the removal of the lesion in its full thickness from the rectum wall, repair of the specimen in a cork piece, and identification of the edges for better evaluation by the pathologist.

In the pathological examination of the tumor, the diameter and distance from the distal edge of the specimen were measured, and all lymph nodes were examined histologically. The pTNM staging of the tumor was done according to the depth of penetration into the rectal wall (pT) and the number of compromised lymph nodes (pN). Complete pathological response was considered when no malignancy was found in the specimen (pT0) or the lymph nodes (pN0). The circumferential resection margin was considered positive when it showed less than 1 mm of the tumor. The grade of pathological tumor regression was based on the scheme proposed by Dworak et al.^17^:

- Grade 0: no sign of regression;
- Grade 1: dominant tumor mass associated with fibrosis and/or vasculopathy;
- Grade 2: dominant presence of fibrosis with few tumor cells isolated or in groups;
- Grade 3: rare tumor cells within fibrotic tissue with or without the concomitant presence of mucin;
- Grade 4: absence of tumor cells, with fibrosis only–total regression.

The outpatient postoperative evolution was assessed every three months during the first two years, with a physical and proctological examination, CEA, chest CT–abdomen every six months, and colonoscopy every year. From the third year onward, appointments every six months, and from the fifth year onward annual CT and colonoscopy.

Local recurrence was defined as the recurrence of the manifestation of the disease inside the pelvis, confirmed by reoperation and surgical resection or biopsy or by finding a lesion in the imaging exams (CT or magnetic resonance imaging) that had an increase in size after some time. Distant recurrence or metastasis was defined as any recurrence outside the pelvic cavity.

The primary endpoint parameters were:

- Overall survival (OS): defined as the interval between the start date of radiation therapy and death from any cause or last appointment;
- Disease-free survival (DFS): defined as the interval between the start date of radiation therapy and the date of detection of any recurrence, either local or distant, or last appointment;
- Metastasis-free survival (MFS): defined as the interval between the start date of radiation therapy and the date of detection of any distant recurrence or last appointment;
- Cancer-specific survival (CSS): defined as the interval between the start date of radiation therapy and death from rectal cancer or the last appointment.

As secondary endpoint parameters, we studied the relationship of OS, DFS, MFS, and CSS with several secondary parameters, which were subdivided for the search for survival-related prognostic factors (Table 1).

**Table 1:** Secondary outcome parameters related to survival.

Parameters		N	%		N	%
Gender	men	37	52.9	women	33	47.1
Age group (years old)	< 56.5	35	50.0	≥ 56,5	35	50.0
Carcinoembryonic antigen (ng/mL)	< 4	31	53.4	≥ 4	27	46.6
Tumor distance from anal verge (cm)	< 5	54	77.1	≥ 5	16	22.9
Radiation dose	45 Gy	37	52.9	50.4 Gy	33	47.1
Grade of tumor clinical regression	Grade 0/ 1	27	38.6	Grade 2/ 3/ 4	43	61.4
Interval radiotherapy and surgery – surgery (weeks)	≤ 8	20	28.6	> 8	50	71.4
Type of surgery	APR	46	65.8	ARR/LRRC	24	34.2
Tumor size (cm)	< 3	34	48.6	≥ 3	36	51.4
TMN staging (pT)	pT0/pT1/pT2	40	57.2	pT3/pT4	30	42.8
TMN staging (pN)	pN0	57	81.4	pN1/pN2	13	18.6
Number of lymph nodes	< 8	23	39.7	≥ 8	35	60.3
TMN staging	0/1	35	50.0	2/3	35	50.0
Grade of Dworak’s pathological tumor response	Grade 0/1/2	45	64.3	Grade 3/4	25	35.7
Circumferential resection margin	Positive	8	11.4	Negative	62	88.6
Complete pathological response	Yes	13	18.6	No	57	81.4

APR: abdominoperineal resection of the rectum; ARR: anterior resection of rectum; LRRC: local resection of rectal cancer. Source: Elaborated by the authors.

### Statistical analysis

Continuous asymmetric and normal distribution variables were described with median (interquartile range) and mean ± standard deviation, respectively. Categorical variables were described with absolute and relative frequencies. Survival curves were calculated using the Kaplan-Meier product-limit method and compared with the Log-rank test when the assumption of proportional hazards was satisfied. Otherwise, the Wilcoxon test was applied.

Survival probabilities were estimated with a 95% confidence interval. Cox univariate analyses were calculated, and Cox multivariate analyses were performed in order to identify factors associated with survival outcomes. All the probabilities of significance (p-values) presented are bilateral. Values below 0.05 were considered statistically significant. Statistical analysis of the data was performed using Statistical Analysis System 9.3 (Cary, NC, United States of America) and R software version 2.15.1.

## Results

The analysis of patient survival has shown that the average follow-up time was 62 months and ranged from 12 to 134 months. In this period, 16 (22.8%) deaths occurred, ten of which (14.2%) were from cancer and six (8.6%) from other causes. The overall recurrence rate was 20% (14 patients). Local recurrence accounted for 4.3%, whereas distant recurrence accounted for 15.7% in the lungs (6), liver (2), bones (2), and adrenal (1).

The 5-year survival analysis has shown that OS was 75.6% (95% confidence interval–95%CI 62.2–84.9, DFS was 76.4% (95%CI 63.1– 85.5), MFS was 77.4% (95%CI 63.8–86.4%), and CSS was 84.4% (95CI% 72–91.6%).

There was no difference in the OS, DFS, MFS, and CSS curves regarding gender, age group, tumor distance from the anal verge, radiation dose used, grade of tumor clinical regression, interval between the end of radiation therapy and surgery, type of surgery, tumor size, number of lymph nodes in the specimen, TNM staging, grade of Dworak’s pathological tumor response, circumferential resection margin and complete or incomplete pathological response.

Regarding the DFS and MFS curves, when the CEA was greater than 4 ng/mL, the evolution was worse in comparison with patients with CEA < 4 ng/mL, with a statistically significant difference.

pT1/pT2 staging in the DFS, MFS, and CSS curves has shown better results than pT3/pT4.

All survival curves revealed that the evolution of patients with malignant lymph node involvement was worse, with a statistically significant difference.

The DFS and MFS curves have shown a statistically significant difference between stages 0 and I concerning stages II and III.

### Research on prognostic variables that influenced patient survival

To identify variables related to survival outcomes, Cox’s univariate and multivariate analysis was used. In the variables related to overall survival by univariate regression, there was no statistically significant correlation. Therefore, multivariate regression was not performed. The variables related to OS, DFS, MFS, and CSS with a statistically significant association in the univariate analysis are shown in Table 2. In multivariate regression, CEA before radiation therapy, pT staging, and pN staging are independent prognostic factors for the patients’ DFS, MFS, and CSS (Table 3).

**Table 2:** Analysis of variables related to survival by univariate Cox regression.

Variables	*p*-value	RR	95%CI	
**Overall survival**				
There was no statistical difference between the variables				
**Disease-free survival**				
CEA < 4 ng/mL × CEA ≥ 4 ng/mL	0.021	3.635	1.214	10.88
pT0/pT1/pT2 × pT3/pT4	0.0069	5.819	1.62	20.896
pN0 × pN1/pN2	0.0036	4.755	1.664	13.585
Staging TNM 0/1 × TNM 2/3	0.0134	6.62	1.481	29.595
**Metastasis-free survival**				
CEA < 4 ng/mL × CEA ≥ 4 ng/mL	0.017	4.21	1.294	13.703
pT0/pT1/pT2 × pT3/pT4	0.0127	5.165	1.419	18.793
pN0 × pN1/pN2	0.0135	3.958	1.329	11.794
Staging TNM 0/1 × TNM 2/3	0.0204	5.952	1.319	26.863
**Cancer-specific survival**				
pT0/pT1/pT2 × pT3/pT4	0.0343	5.345	1.132	25.245
pN0 × pN1/pN2	0.018	4.471	1.293	15.462

CEA: carcinoembryonic antigen; RR: relative risk; 95%CI: 95% confidence interval. Source: Elaborated by the authors.

**Table 3:** Analysis of variables related to DFS, MFS and CSS by Cox multivariable regression.

Variables	*p*-value	RR	95%CI	
**Disease-free survival**				
CEA < 4 ng/mL × CEA ≥ 4 ng/mL	0.0235	3.645	1.19	11.161
pT0/pT1/pT2 × pT3/pT4	0.0162	4.827	1.338	17.411
pN0 × pN1/pN2	0.0028	5.174	1.759	15.224
**Metastasis-free survival**				
CEA < 4 ng/mL × CEA ≥ 4 ng/mL	0.0169	4.302	1.3	14.234
pT0/pT1/pT2 × pT3/pT4	0.0317	4.129	1.132	15.051
pN0 × pN1/pN2	0.0118	4.141	1.371	12.511
**Cancer-specific survival**				
pT0/pT1/pT2 × pT3/pT4	0.0134	5.086	1.402	18.449
pN0 × pN1/pN2	0.0105	3.994	1.384	11.526

CEA: carcinoembryonic antigen; RR: relative risk; 95%CI: 95% confidence interval; CSS: cancer-specific survival; MFS: metastasis-free survival; DFS: disease-free survival. Source: Elaborated by the authors.

## Discussion

In the treatment of rectal cancer, surgery with excision of the mesorectum is the most important therapeutic modality, but the use of radiation therapy associated or not with chemotherapy in the preoperative period has been well studied in the literature.

The Kapiteijn et al.^11^ and Swedish Rectal Cancer Trial et al.^18^ compared preoperative radiation therapy followed by surgery with surgery alone. In the first study, no improvement in overall survival was found, but less local recurrence in the irradiated group. In the second study, there were reduction in local recurrence (27–11%) and increase in survival (overall 48–58%).

To compare the preoperative RCT with that performed in the postoperative period, Sauer et al.^13^ studied 823 patients in two groups with preoperative and postoperative RCT. There was no difference in overall or disease-free survival in five years. The rate of local recurrence was lower in the group that received preoperative radiation therapy.

Habr-Gama et al.^19^, in a sample of 183 patients who received preoperative RCT and a watch-and-wait strategy, found a complete tumor response rate of 49%. The five-year cancer-specific overall survival and disease-free survival for all patients (including all recurrences) were 91 and 68%, respectively.

The synergistic effect of preoperative radiation therapy when combined with chemotherapy was demonstrated in three studies. Boulis-Wassif et al.^20^, when comparing two groups with 247 patients with isolated RT and preoperative RCT, found better overall survival in the group that received CT (59 vs. 46%). Gerard et al.^21^ studied 742 patients and found no difference in overall or disease-free survival. However, patients with RT alone had a higher rate of local recurrence, a lower rate of complete pathological response of the tumor, and acute toxicity to the neoadjuvant treatment. Erlandsson et al.^22^ demonstrated that the use of RT and CT allows waiting for surgery of four to eight weeks and is oncologically safe.

Valenti et al.^23^ did not find differences regarding postoperative complications and mortality in patients who received preoperative RCT and those who received surgery only.

Latkauskas et al.^24^ and Abraha et al.^25^ carried out a systematic literature review and meta-analysis of preoperative chemoradiotherapy compared to exclusive RT or surgery and concluded that preoperative chemoradiotherapy in stage-II and III rectal cancer produces a higher rate of complete pathological tumor response and reduces the rate of local recurrence.

The data in Table 4 show the mean follow-up time, the recurrence rate, and the survival rates of different studies in the literature.

In this research, the mean follow-up was 62 months. The follow-up time can be considered high compared to the literature. This reflects an adequate long-term outpatient follow-up, which reinforces the findings in terms of overall survival related to recurrence, metastases, and cancer-specific in the studied group.

**Table 4:** Mean follow-up time, recurrence rate and survival rate of studies in the literature.

Authors	N	MFU	RR	OS	DFS	MFS	CSS
Yeo et al.^7^	364	68	21,9	87,2	78,8	81,6	NR
Fokas et al.^10^	1,179	50	3,2*	88,4	73,6	NR	NR
Habr-Gama et al.^19^	183	60	31,0	NR	68,0	NR	91
Valentini et al.^26^	165	67	NR	74,0	63,0	NR	NR
Pucciarelli et al.^27^	106	42	9,7	83,6	83,6	NR	NR
Hughes et al.^28^	211	25	18,0*	NR	NR	NR	NR
Lindebjerg et al.^29^	135	26	8,9	NR	NR	NR	82,0
Kim et al.^30^	178	52	14,6	NR	NR	NR	72,3
Ha et al.^31^	615	58	NR	82,3	76,8	NR	NR
Kim TH et al.^32^	420	50,5	22,9	88,7	75,4	77	NR
Kong et al.^33^	135	50	22,1*	82,7	NR	67,9	NR
Martens et al.^34^	100	41,1	15,0	96,6	80,6	84,6	NR
Smith et al.^35^	249	43	20,0	73,0	75,0	NR	90,0
Hupkens et al.^36^	292	58	14,3	97,1	NR	94,3	NR
Jimenez-Rodriguez et al.^37^	88	42	18,2	NR	NR	NR	NR

MFU: mean follow-up time in months; RR: recurrence rate (%); *local recurrence; OS: overall survival (%); DFS: disease-free survival (%); MFS: metastasis-free survival (%); CSS: cancer-free survival (%); NR: not referred. Source: Elaborated by the authors.

The rate of tumor recurrence pointed out by the authors ranged from 8.9 to 31%. The recurrence in 14 (20%) patients in this study fell within the range presented by the different authors.

Regarding the survival rates in this study, the OS in five years was 75.6%, the DFS was 76.4%, the MFS was 77.4%, and the CSS was 84.4%. When comparing them with the rates presented by the different studies, it was found that they were comparable.

CEA is used to diagnose recurrence in the postoperative period. When measured before surgery, it can be used as a prognostic indicator. Comparing CEA results before and after neoadjuvant therapy, Perez et al.^38^ concluded that, when this marker is below 5 ng/mL after radiation therapy, patients have an increase in the rate of complete clinical and pathological response, in addition to improvement in OS and DFS. This study compared patients with CEA lower than 4 ng/mL and those with a level equals to or higher than that in a period before the RCT. There was a statistically significant difference between the DFS and MFS curves concerning the two groups, as well as in the univariate analysis. Multivariate regression proved that CEA is an independent prognostic factor for DFS and MFS.

With the possibility of regression of the invasion of the rectal wall by the tumor and the sterilization of the compromised lymph nodes (downstaging), experiences of local resection of rectal tumor treated with preoperative chemotherapy and radiation therapy began to be published. Criticism of this operative conduct concerns the lack of accessibility to lymph nodes that may be involved. Creavin et al.^39^ compared 50 individuals with local resection with 362 from whom the mesorectum was excised. The authors found no differences in overall survival or disease-free survival rates between the two groups. In this study, 12 local resection surgeries were performed for rectal tumors. The survival of these patients was compared to that of patients who underwent surgeries with mesorectum excision, such as lower anterior resection. The authors found no differences in OS, DFS, MFS, and CSS between these two types of operative treatment.

The pT staging was an independent factor in multivariate regression for OS and DFS, which was observed by Dulk et al.^40^. In the multivariate analysis done by Hwang et al.^41^, the degree of pT staging was an independent prognostic factor for DFS. In this research, the pT0, pT1, and pT2 staging groups presented DFS, MFS, and CSS curves with a statistically significant difference with pT3 and pT4. Likewise, pT staging proved to be an indicative prognostic variable in univariate analysis and an independent prognostic factor in multivariate regression for DFS, MFS, and CSS.

Lymph node involvement by the cancer is the most important prognostic factor for long-term outcomes. Lee et al.^42^ described pN as a variable related to OS and DFS in the univariate analysis and OS in the multivariate. Huebner et al.^43^ found that lymph node involvement was associated with DFS and CSS in multivariate regression. Cho et al.^44^ demonstrated the five-year recurrence-free survival and OS in patients with lymph node metastasis were not favorable compared with those of N0 disease. In the results obtained with the patients in this study, the importance of the pN variable as a prognostic factor was clear since it proved statistically significant in the curves of OS, DFS, MFS, and CSS, as well as in the univariate and multivariate regression for DFS, MFS, and CSS.

The staging was considered a variable related to DFS, both in the univariate analysis and the multivariate regression, and it is an independent prognostic factor for recurrence, according to a study by Kim et al.^32^. Hayes et al.^45^ did not find a relationship between downstaging of the locally advanced rectal cancer after neoadjuvant chemoradiotherapy with reduced recurrence. In this study, the comparison of the two groups (grades I and II × III and IV) revealed that there was a statistically significant difference between DFS and MFS. This result was confirmed in univariate regression, expressing that the more advanced the stage is, the worse the prognosis is for survival in terms of recurrence in general and distant recurrence in particular.

The tumor response to radiation therapy appeared as an independent prognostic factor for DFS and OS because patients with complete pathological response had a significantly greater DFS and OS than non-responders^46^^,^ 47. However, this research did not identify the influence of the tumor pathological regression variable on the survival outcomes of this series.

Sterilization of the original tumor of the rectum occurs at rates ranging from 8.8 to 22%. In this series, the complete pathological response of the tumor was 18.6%. Although there is a significant relationship between this result and DFS and OS, as demonstrated by Lee et al.^48^ and Sell et al.^49^, this study could not confirm the beneficial effect of the complete pathological response on the evolution of patients.

## Conclusion

Preoperative radiation therapy and chemotherapy have been demonstrated to be safe and effective treatments, contributing to favorable recurrence and survival rates in line with existing literature. Notably, elevated levels of CEA above 4 ng/mL before initiating radiation therapy were associated with poorer outcomes. Additionally, patients with pT3 and pT4 stages exhibited shorter survival compared to those with pT0, pT1, and pT2 classifications. Similarly, pN1 and pN2 staging corresponded with reduced survival rates compared to patients categorized as pN0. These findings indicate that pre-treatment CEA levels, pT staging, and pN staging are independent prognostic factors influencing survival in patients with rectal adenocarcinoma undergoing preoperative radiation therapy.

## Data Availability

Data sharing is not applicable.
